# Age and geographic patterns of *Plasmodium falciparum* malaria infection in a representative sample of children living in Burkitt lymphoma-endemic areas of northern Uganda

**DOI:** 10.1186/s12936-017-1778-z

**Published:** 2017-03-20

**Authors:** Marlena Maziarz, Tobias Kinyera, Isaac Otim, Paul Kagwa, Hadijah Nabalende, Ismail D. Legason, Martin D. Ogwang, Samuel Kirimunda, Benjamin Emmanuel, Steven J. Reynolds, Patrick Kerchan, Moses M. Joloba, Andrew W. Bergen, Kishor Bhatia, Ambrose O. Talisuna, Robert J. Biggar, James J. Goedert, Ruth M. Pfeiffer, Sam M. Mbulaiteye

**Affiliations:** 1 Division of Cancer Epidemiology and Genetics, National Cancer Institute, National Institutes of Health, Bethesda, MD USA; 2 EMBLEM Study, African Field Epidemiology Network, Kampala, Uganda; 3 St. Mary’s Hospital, Lacor, Gulu, Uganda; 4 Department of Medical Microbiology, Makerere Medical School, Kampala, Uganda; 5 University of Maryland, Baltimore, MD USA; 6 Division of Intramural Research, National Institute of Allergy and Infectious Diseases, National Institutes of Health, Bethesda, MD USA; 7 Kuluva Hospital, Kuluva, Arua, Uganda; 8 World Health Organization, Regional Office for Africa, Brazzaville, Congo; 9 Institute of Health and Biotechnical Innovation, Queensland University of Technology, Brisbane, Australia

**Keywords:** Burkitt lymphoma, Africa, *Plasmodium falciparum*, Malaria, Epidemiology, Non-Hodgkin lymphoma, Uganda

## Abstract

**Background:**

Falciparum malaria is an important risk factor for African Burkitt lymphoma (BL), but few studies have evaluated malaria patterns in healthy BL-age children in populations where both diseases are endemic. To obtain accurate current data, patterns of asymptomatic malaria were investigated in northern Uganda, where BL is endemic.

**Methods:**

Between 2011 and 2015, 1150 apparently healthy children under 15 years old were sampled from 100 villages in northern Uganda using a stratified, multi-stage, cluster survey design. Falciparum malaria prevalence (pfPR) was assessed by questionnaire, rapid diagnostic test (RDT) and thick film microscopy (TFM). Weighted pfPR and unadjusted and adjusted associations of prevalence with covariates were calculated using logistic models and survey methods.

**Results:**

Based on 1143 children successfully tested, weighted pfPR was 54.8% by RDT and 43.4% by TFM. RDT sensitivity and specificity were 97.5 and 77.8%, respectively, as compared to TFM, because RDT detect malaria antigens, which persist in peripheral blood after clinical malaria, thus results based on RDT are reported. Weighted pfPR increased from 40% in children aged under 2 years to 61.8% in children aged 6–8 years (odds ratio 2.42, 95% confidence interval (CI) 1.26–4.65), then fell slightly to 49% in those aged 12–15 years. Geometric mean parasite density was 1805.5 parasites/µL (95% CI 1344.6–2424.3) among TFM-positive participants, and it was higher in children aged <5 years at 5092.9/µL (95% CI 2892.7–8966.8) and lower in those aged ≥10 years at 983.8/µL (95% CI 472.7–2047.4; *P* = 0.001). Weighted pfPR was lower in children residing in sub-regions employing indoor residual spraying (IRS) than in those residing in non-IRS sub-regions (32.8 versus 65.7%; OR 0.26, 95% CI 0.14, 0.46). However, pfPR varied both within IRS (3.2–55.3%) and non-IRS sub-regions (29.8–75.8%; *Pheterogeneity* <0.001). pfPR was inversely correlated with a child’s mother’s income (P = 0.011) and positively correlated with being enrolled in the wet season (P = 0.076), but sex was irrelevant.

**Conclusions:**

The study observed high but geographically and demographically heterogenous patterns of asymptomatic malaria prevalence among children living in northern Uganda. These results provide important baseline data that will enable precise evaluation of associations between malaria and BL.

**Electronic supplementary material:**

The online version of this article (doi:10.1186/s12936-017-1778-z) contains supplementary material, which is available to authorized users.

## Background

Falciparum malaria is an important cause of childhood morbidity and mortality in young children in Equatorial Africa [1, 2]. It is also an important risk factor for African Burkitt lymphoma (BL) [3], a B cell tumour that is the most common cancer in children in many countries in Equatorial Africa [4], including Uganda. The association of falciparum malaria with BL is based on the geographical correlation of high incidence of severe malaria and high incidence of BL [5–7], and on the significant positive or inverse association of high titres of particular malaria antibodies in children with BL [8–10]. The epidemiological evidence is supported by evidence from mouse studies, which have shown that infection of mice with *Plasmodium chabaudi* increases the risk of genetic abnormalities in their B cells, including those similar to the characteristic abnormalities seen in BL involving the translocation of exons in the *MYC* gene on chromosome 8 into the vicinity of enhancer or promoter regions for immunoglobulin genes on chromosomes 14, 2, or 22 [11, 12].

Whether clinical malaria or the significantly more prevalent asymptomatic parasitaemia similarly affect BL risk is unknown [3]. The overlap between severe malaria and BL by age is imperfect [1, 2] because severe malaria occurs most commonly in young children of around 2 years old [13], whereas BL peaks in children around 6–9 years old [14, 15]. Since BL is an extremely fast-growing tumour [16], the imperfect overlap between the peak ages for severe malaria and BL does not support the idea that severe malaria is the antigenic insult that triggers BL onset. This reasoning is supported by the casual observations that a history of severe malaria is not a commonly elicited feature in children with BL suggesting that older children in the age range of BL are protected against severe malaria by acquired immunity [17, 18] in agreement with findings that the probability of clinical malaria falls by 6% per year of age and by 2% per episode of clinical malaria [19]. Thus, the timing of peak BL risk is consistent with recurrent mild or asymptomatic malaria parasitaemia being the malaria insults that triggers the onset of the genetic abnormalities that lead to BL.

Clarifying the malaria phenotype that is relevant for BL aetiology is an important question that remains unanswered because research efforts are hampered by the lack of reliable data about malaria experience among children of a similar age and sex distribution as BL cases living in BL-endemic areas. To address this knowledge gap, detailed epidemiological data and biological samples were collected from a representative population-based sample of healthy children from northern Uganda with a similar age and sex distribution as typical BL cases. This area has historically experienced holoendemic malaria [20–22] (weighted *Plasmodium falciparum* parasite prevalence (pfPR) over 75%) [22] and high incidence of BL [7]. Baseline data from field-collected data were analysed to obtain a more granular picture of the geographical and demographical patterns of asymptomatic malaria infection in this region. This would pave the way for follow-up studies conducted using molecular and immunological methods to evaluate the malaria response in patients with and without BL and to perform more precise adjustment of the geographical and demographical co-factors.

## Methods

### The aim, design and setting of the study

To obtain geographically and demographically representative data about malaria in northern Uganda, between January 2011 and April 2015, apparently healthy children (0–15 years of age) from the northwest and north-central regions of Uganda were enrolled as part of the EMBLEM study of BL [23]. These two regions were selected because they have historically experienced holoendemic malaria transmission [20] and they experience a correspondingly high, albeit geographically variable, BL endemicity [7]. The annual entomological inoculation rate (AEIR) varies from 397 in  the Arua District in the northwest region to 1586, the highest number recorded worldwide, in  the Apac District in the north-central region [24]. Between 2009 and 2012, the Government of Uganda responded to this high AEIR by implementing indoor residual spraying (IRS) of insecticides and distributing insecticide-treated bed nets to pregnant women and children under 5 years old in 10 districts in the north-central region, hereafter called the IRS sub-region [21]. The non-sprayed area is called the non-IRS sub-region.

### Sampling design

**Fig. 1:**
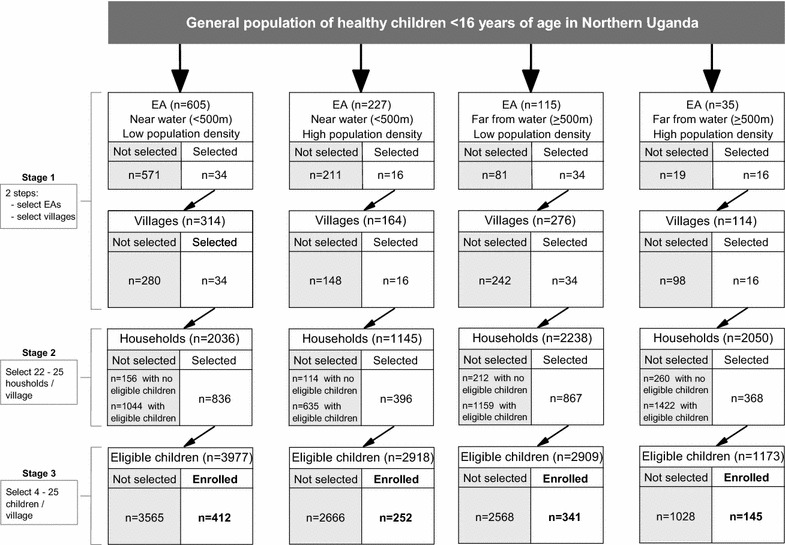
Flow chart showing the sampling of healthy children aged 0–15 years from 100 villages in north-central and northwest regions of Uganda between January 2011 and April 2015

A stratified multi-stage cluster sampling design was used to select a regionally representative, population-based sample of healthy children ages 0–15 years old from was selected 100 villages in the study region (Fig. 1). To select the villages, 100 census enumeration areas (EAs) were selected from a complete list of EAs in the study region obtained from the Uganda Bureau of Statistics (UBOS) [25]. This EA sample frame was stratified on ‘low-density’ versus ‘high-density’ population and ‘near water’ versus ‘far from water’ before sampling. Since the stratifying variables are thought to influence mosquito ecology and through that malaria transmission [26, 27], they were considered domains of special interest when estimating geographical patterns of malaria. Low-density EA stratum, considered a surrogate for rural areas, was defined as including those EAs having a population count of children younger than 15 years fewer than the EA mean population count (n = 2683) [25]; otherwise the EAs were categorized as belonging to a high-density EA stratum, considered a surrogate for urban areas. Near water EA stratum was defined as including those EAs with a boundary next to or within 500 m of an all-season surface water body (a swamp, river, lake), based on distances estimated from national maps incorporating geographical information metadata; otherwise the EAs were categorized as belonging to a far from water EA stratum. Because 90% of the Ugandan population resides in rural (or low-density) areas, the high-density EA stratum were oversampled by selecting low-density: high-density EAs in a ratio of 2:1. Thus, 68 EAs were sampled from low-density strata and 32 EAs from high-density with equal probability in each stratum, maintaining a ratio of 1:1 for near water versus far from water EAs within each stratum.

Because the EAs are large geographical areas that were logistically challenging for research staff to accurately survey, they were segmented into four to 17 units per EA using existing local administrative units called Local Council 1 (LC-1), also known as villages and that have distinct non-overlapping boundaries, as segments. One segment was randomly selected with equal probability per EA. Thus, one segment or village represented the entire EA. In the next stage, 22–25 households per segment were selected with equal probability from a household list constructed by study staff. In the third stage, four to 25 children were selected from a list of all children in the selected households in that segment, based on residing in the study region for at least 4 months and according to the predetermined age and sex frequency distribution of typical BL cases in the region [7].

### Participant enrolment

Experienced fieldwork teams visited participants in their homes and invited them to participate in the study after obtaining informed consent. Structured questionnaires were used to collect participant information about the demographics and household characteristics, educational level of a child’s parents, a child’s exposure to malaria suppression methods (use of mosquito bed nets the night before interview and use of IRS in the house), and history of outpatient or inpatient malaria treatment (up to 6 months ago, 7–12 months ago, or 13 or more months ago). A venous blood sample for immediate malaria testing and for storage for future studies was obtained from the participant child using EDTA tubes.

### Malaria testing

Malaria infection was diagnosed by experienced laboratory technicians using thick film microscopy (TFM) to identify asexual malaria parasite forms in thick film slides stained with 10% Giemsa solution for 10 min. Malaria parasites were counted against 200 white blood cells (WBCs) and standardized to parasites/µL of blood based on the measured WBC/µL. Thin film smears were examined to identify *Plasmodia* species. Since ~98% were *P. falciparum*, this species is assumed hereafter. The technicians also used antigen–antibody capture rapid diagnostic tests (RDT) (MALARIA DUAL kits, ICT Diagnostics, Muizenberg, Cape Town, South Africa) to diagnose malaria infection. These kits detect the *P. falciparum*-specific malaria histidine-rich protein 2 (*Pf*-HRP2) and the pan-lactate dehydrogenase (pLDH) antigen shared by other *Plasmodia* that parasitize humans. The sensitivity and specificity of the kits for malaria in clinical samples in Uganda was reported to be 92–100% [28], which is adequate for the objectives of assessing epidemiological patterns of malaria in the current study. As only 2.1% of the children studied had fever at the time of enrolment, this number was considered negligible and unlikely to impact the overall results so these children were not excluded. The children with fever and positive RDT or TFM were treated for possible clinical malaria with artemisinin-based combination therapy (ACT) as first line drug for treatment of mild clinical malaria following national treatment guidelines. Parents of children without symptoms were advised to seek treatment from their local health centre if a new fever developed.

### Data management

Questionnaires were edited in the field and computerized using DataFax and the data submitted via a secure share-portal to Information Management Services, Inc. (IMS, Calverton, MD, USA) to generate analysis files.

### Statistical analysis

Descriptive analyses of pfPR, based on RDT, by geographical and demographical characteristics of participants are reported. To obtain regionally representative results, prevalence estimates were calculated as weighted probabilities of selecting the children in the study sample using standard survey statistical packages [29, 30] implemented in R (version 3.2.3). Details of the calculation of sampling weights are described in Additional file 1: Appendix 1 and 2. Thus, the reported estimates are representative for all the children in this region and the variance estimation took the weights into account and accounted for the clustering of the sample of children in the segments (villages). Unadjusted and adjusted odds ratios (ORs), standard errors [31] and Wald-type 95% CIs (95% CIs) [32] of association of pfPR with each variable were calculated. The adjusted models included only those variables with *P* < 0.10. Because parasite load in asymptomatic children is partly a function of cumulative acquired immunity to malaria, the overall geometric mean parasite density (GMPD) per µL were calculated and compared by sex and age using the Student’s t test statistic. As the objective of these analyses was to describe patterns to obtain granular baseline data for hypothesis generation, the results were not adjusted for multiple testing. Thus, a two-sided *P* < 0.05 was considered as evidence of statistical significance and values *P* < 0.10 as suggesting a trend. Analyses of malaria patterns were repeated separately using pfPR defined based on only TFM and on both TFM and RDT and similar results were obtained.

## Results

### Characteristics of study population

**Fig. 2:**
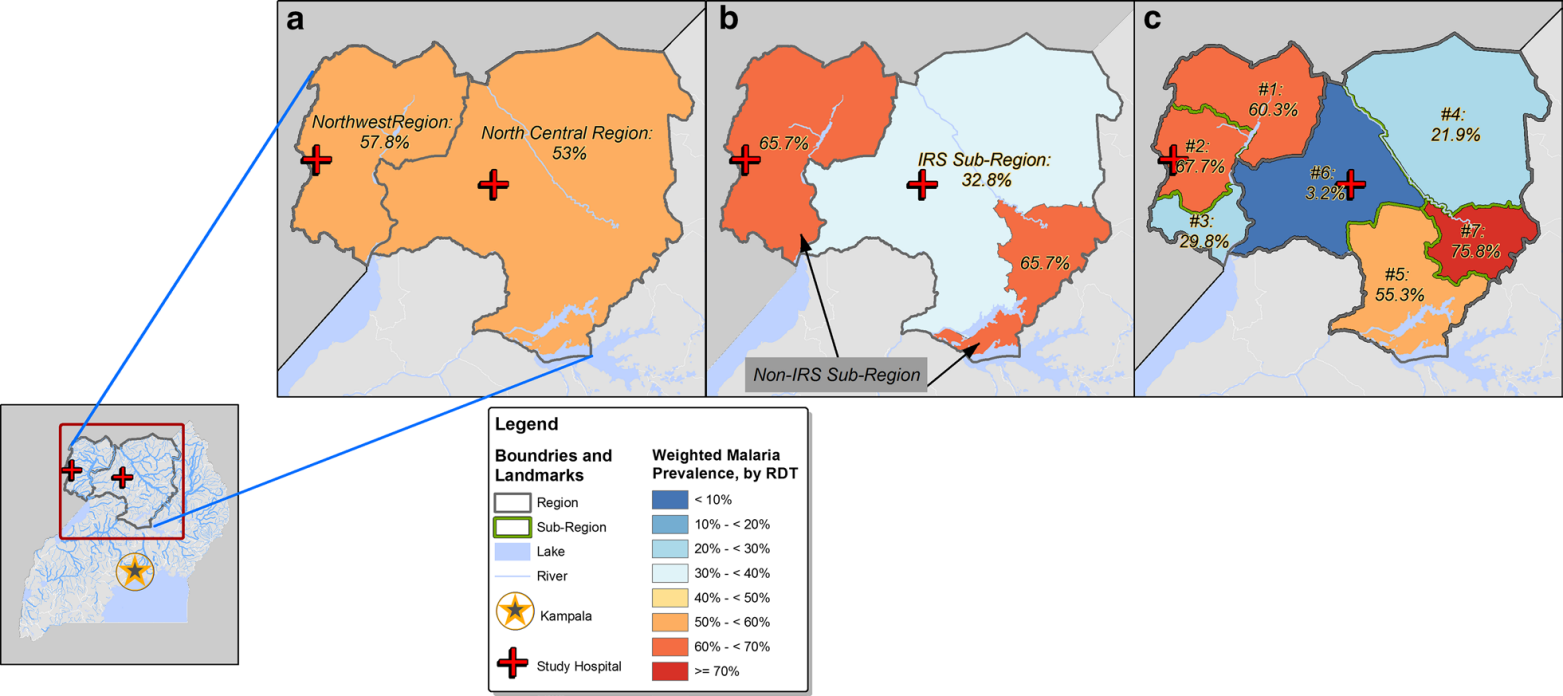
Map of north-central and northwest regions in Uganda. Showing weighted *Plasmodium falciparum* parasite prevalence based on the rapid diagnostic test in a representative sample of 1142 healthy children 0–15 years old enrolled between January 2011 and April 2015. *Panel* **a** pfPR patterns in the two north-central and northwest regions; *Panel* **b** pfPR patterns in two sub-regions employing or not IRS; *Panel* **c** pfPR patterns in seven sub-regions. A *inset* map is included to show the location of north-central and northwest regions in relation to Kampala, the capital city of Uganda, and the location of collaborating hospitals in the study area. It also shows major Ugandan water features (rivers and lakes)

Overall, 2484 households were selected in 100 villages and 2467 of them participated. From these households, 1167 children were invited to participate and 1150 of them were enrolled, representing a weighted sample of 585,762 children in the region. Consistent with the design, to select children with a typical age and gender distribution typical of BL, 44.6% children participating were 6–10 years old and 52.1% were males (Table 1). Consistent with the predominantly rural population of northern Uganda, 67.6% of the children lived in low-density villages and 89.3% lived in villages near water. By region, 60.8% were from the north-central region and 39.2% from the northwest region (the location of these regions is shown in Fig. 2a). With respect to malaria suppression, 32.9% lived in the sub-region employing IRS and 67.1% in the non-IRS sub-region (as defined in Fig. 2b). To further explore malaria variation in sub-regions, the study area was sub-divided into seven sub-regions based on boundaries that correspond roughly to tribal areas (as shown in Fig. 2c). Two of these sub-regions, which contribute to the majority of BL cases in this region [7], contributed 50% of the study population. Some 28.9% of the children reportedly used a mosquito bed net on the night before interview, but 31.3% lived in houses using IRS, which is similar to the per cent living in an IRS sub-region. Regarding socio-economic status, 55.3% of mothers of participants had fewer than 5 years of formal education and 55.7% had a monthly median income below 30,000 Ugandan shillings (equivalent to ~US$10). Regarding history of malaria, 63.5% of the participants reported a history of outpatient malaria treatment, with 55.6% reporting such treatment in the past 12 months. Conversely, only 36.6% reported at least one inpatient treatment for malaria, defined as requiring some observation at a clinic or hospital, with 12.6% reporting such treatment in the past 12 months.

**Table 1:** Weighted distribution of characteristics of apparently healthy children aged 0–15 years enrolled between January 2011 and April 2015 from the north-central and northwest regions of Uganda

	N = 1150	Weighted % (95% CI)
Age group, years		
0–5	326	28.0 (25.0–31.0)
6–10	572	44.6 (41.6–47.7)
11–15	252	27.4 (23.7–31.1)
Sex		
Female	541	47.9 (44.4–51.4)
Male	609	52.1 (48.6–55.6)
Proximity to water		
Far (>500 m)	486	10.7 (8.1–13.4)
Near (≤500 m)	664	89.3 (86.6–91.9)
Population density		
Low (<2683 children)	753	67.6 (60.0–75.2)
High (≥2683 children)	397	32.4 (24.8–40)
Season		
Dry season	731	69.9 (57.7–82.2)
Wet season	419	30.1 (17.8–42.3)
Region		
North-central	697	60.8 (46.9–74.8)
Northwest	453	39.2 (25.2–53.1)
Indoor residual spraying (IRS) sub-region		
Not an IRS district	737	67.1 (53.1–81.0)
IRS district	413	32.9 (19.0–46.9)
Indoor residual spraying (IRS) in house		
More than a year ago	752	68.7 (55.8–81.6)
In the past year	392	31.3 (18.4–44.2)
Sub-regions		
1	113	7.9 (2.6–13.2)
2	289	22.6 (10.5–34.6)
3	51	8.7 (0.5–17.9)
4	97	9.6 (2.1–17.1)
5	303	34.5 (20.0–49.0)
6	90	2.3 (0.1–4.4)
7	207	14.5 (4.5–24.5)
Mother’s education		
Up to primary 4	581	55.3 (49.5–61.0)
Primary 5 or higher	565	44.7 (39.0–50.5)
Mother’s income (Ugandan shillings)		
<30,000 USHS	560	55.7 (48.8–62.6)
≥30,000 USHS	585	44.3 (37.4–51.2)
Mosquito net used last night		
No	777	71.1 (63.4–78.8)
Yes	366	28.9 (21.2–36.6)
Inpatient malaria		
No	725	63.4 (57.3–69.5)
Past 12 months	150	12.6 (9.2–16.0)
More than 12 months	269	24 (17.7–30.3)
Outpatient malaria		
No	523	36.5 (28.5–44.5)
Past 12 months	532	55.6 (47.5–63.6)
More than 12 months	89	7.9 (5.6–10.3)

N shows unweighted numbers; totals in some categories may not add up to 100% because of missing data. Dry season months were January to March and July to August; Wet season months were April to June and September to December. Mother’s income was estimated in Ugandan shillings (30,000 Ugandan shillings are approximately equal to 10 US dollars). The survey estimates are weighted estimates that account for the differential probabilities in selecting the sample of children. Variance estimation takes the weights into account and also accounts for the clustering of the sample of children at the village level. The coefficient of variation of the final weights was 1.27 (defined as standard deviation/mean of the final weights)

### Weighted malaria prevalence and density

Malaria testing was successful for 1143 children; the seven children who failed were excluded from further analysis (Table 2). The weighted pfPR based on RDT was 54.8% (95% CI 47.3–62.4%) and it was 43.4% (36.1–50.7%) based on TFM. As expected, the sensitivity of RDT was 97.5% and the specificity was 77.8%, compared to TFM. RDT is more sensitive than TFM because it detects malaria antigens, which can be detected during early infection and for up to 35–42 days after treatment of symptomatic cases due to persistence of antigens in peripheral blood [33]. Using positivity to both RDT and TFM to define current parasitaemia, the weighted pfPR of current parasitaemia was 42.3%. Conversely, using only RDT positivity to define malaria antigenaemia alone (due to early or persistent antigen after infection has resolved), the weighted pfPR of malaria antigenaemia alone was 12.6%. The weighted prevalence of negative RDT among TFM-positive subjects (including two with parasitaemia identified only on thin film) was 1.1%. These cases (TFM-positive and RDT-negative) had lower GMPD of 178.5 parasites/µL (95% CI 115.0–277.0) than those with of current infection (RDT and TFM positive) with GMPD of 1912.3 parasites/µL (95% CI 1423.0–2570.0; *P* < 0.001).

**Table 2:** Weighted *Plasmodium falciparum* parasite prevalence according to the rapid diagnostic test and thick film microscopy among healthy children in northern Uganda

		Thick film microscopy		Total
		Negative	Positive	
RDT	Negative	44.1% (n = 561)	1.1% (n = 12)	45.2% (n = 573)
	Positive	12.6% (n = 141)	42.3% (n = 429)	54.8% (n = 570)
	Total	56.6% (n = 702)	43.4% (n = 441)	100% (N = 1143) (N_weighted_ = 585,762)

Seven subjects who failed malaria testing were excluded from further analysis. The percentages in each cell are weighted back to the population of size 585,762; the numbers in parentheses are the numbers of individuals those percentages are based on

### Geographical and seasonal variation in weighted pfPR

**Fig. 3:**
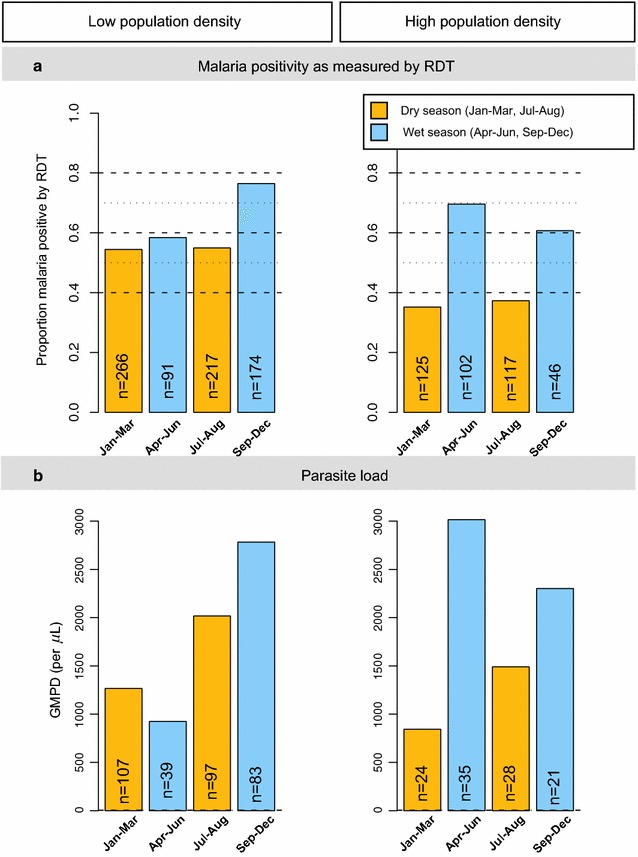
*Bar graphs* showing malaria weighted per cent *Plasmodium falciparum* parasite prevalence. Based on the RDT, by season (wet or dry) in low- and high-population density villages (*Panel* **a**) and the GMPD/µL among microscopy-positive children by visit season in low- and high-population density villages (*Panel* **b**) among apparently healthy children enrolled between January 2011 and April 2015 from north-central and northwest Uganda.* Orange shading* is used for dry season months, while *blue shading* is used for wet season months. Wet and dry seasons based on categorization by the Uganda Bureau of Statistics and generally corresponding to ≥10 days/month for wet months and <10 days/month for dry months. The unweighted number of participants in each group is shown

Similar to prior studies from this region [20, 34, 35], there was no difference in the weighted pfPR estimate for the north-central and northwest regions (Fig. 2a). However, these similar malaria patterns masked variation in sub-regional analysis. First, pfPR was a substantially lower in IRS than non-IRS sub-regions (32.8 versus 65.7%; Fig. 2b). However, the apparent homogeneity in IRS and non-IRS sub-regions masked further, substantial variation in sub-regions in the IRS (3.2–55.3%) and the non-IRS sub-regions (55.5–75.8%; Fig. 2c). This additional variation was not explained by patterns in other covariates (Table 3). As expected, the weighted pfPR (Fig. 3a) and the GMPD (Fig. 3b) showed strong seasonal effects, both being higher during the wet season than the dry seasons, but this pattern was more striking in the high-density than in the low-density villages.

**Table 3:** *Plasmodium falciparum* parasite prevalence among apparently healthy children 0–15 years old enrolled between January 2011 and April 2015 from the north-central and northwest regions of Uganda

Characteristics		Unadjusted		*P*	Adjusted*	*P*
		Weighted (%)	Odds ratio (95% CI)		Odds ratio (95% CI)	
All subjects	1142	54.8	–	–	–	–
Age group, years						
0–5	326	52.0	1.00			
6–10	572	58.2	1.29 (0.86–1.93)			
11–15	252	52.3	1.01 (0.56–1.85)	0.185	–	–
Sex						
Female	541	55.2	1.00			
Male	609	54.5	0.97 (0.68–1.39)	0.885	–	–
Proximity to water						
Far (>500 m)	486	51.6	1.00			
Near (≤500 m)	664	55.2	1.16 (0.63–2.12)	0.631	–	–
Population density						
Low (<2683 children)	753	59.1	1.00			
High (≥2683 children)	397	46.0	0.59 (0.29–1.21)	0.151	–	–
Season						
Dry season	731	48.7	1.00		1.00	
Wet season	419	69.1	2.35 (1.26–4.40)	*0.009*	1.81 (0.95–3.46)	*0.076*
Region						
North-central	697	53.0	1.00			
Northwest	453	57.8	1.21 (0.65–2.28)	0.547	–	–
Indoor residual spraying (IRS) sub-region						
Not an IRS district	737	65.7	1.00			
IRS district	413	32.8	0.26 (0.14–0.46)	<*0.001*	–	–
Indoor residual spraying (IRS) in house						
More than a year ago	752	64.4	1.00		1.00	
In the past year	392	34.0	0.28 (0.15–0.53)	<*0.001*	0.37 (0.14–1.03)	*0.061*
Sub-regions						
1	113	60.3	1.00		1.00	
2	289	67.7	1.38 (0.68–2.78)		1.30 (0.66–2.57)	
3	51	29.8	0.28 (0.07–1.15)		0.25 (0.07–0.86)	
4	97	21.9	0.18 (0.08–0.45)		0.43 (0.11–1.62)	
5	303	55.3	0.81 (0.39–1.69)		1.10 (0.44–2.75)	
6	90	3.2	0.02 (0.00–0.10)		0.03 (0.01–0.24)	
7	207	75.8	2.06 (0.77–5.52)	<*.001*	1.12 (0.43–2.89)	*0.001*
Mother’s education						
Up to primary 4	581	53.6	1.00			
Primary 5 or higher	565	56.4	1.12 (0.78–1.60)	0.551	–	–
Mother’s income (Ugandan shillings)						
<30, 000 USHS	560	59.0	1.00 (1.00–1.00)		1.00	
≥30,000 USHS	585	49.6	0.68 (0.47–1.00)	*0.055*	0.57 (0.37–0.87)	*0.011*
Mosquito net used last night						
No	777	54.2	1.00			–
Yes	366	56.4	1.09 (0.64–1.87)	0.743	–	
Inpatient for malaria						
No	725	54.2	1.00			
Past 12 months	150	42.8	0.63 (0.35–1.15)			
More than 12 months	269	63.1	1.45 (0.88–2.39)	0.119	–	–
Outpatient for malaria						
No	523	46.9	1.00		1.00	
Past 12 months	532	60.4	1.73 (1.10–2.72)		1.28 (0.82–2.01)	
More than 12 months	89	52.6	1.26 (0.53–2.99)	*0.064*	1.00 (0.37–2.69)	0.476

Dry season months were January to March and July to August; Wet season months were April to June and September to December. Mother’s income was estimated in Ugandan shillings (30,000 Ugandan shillings are approximately equal to 10 US dollars). Final adjusted models included all variables with P < 0.10 (season, IRS of insecticide, sub-region, mother’s income, and outpatient malaria treatment). IRS in the sub-region was not included in the model despite its unadjusted P < 0.001, since it was highly correlated with IRS in the house

Results with a two-sided P < 0.05 were considered statistically significant and those with P < 0.10 were considered as showing a trend towards significance. These results are shown in italics. * The adjusted models were fit including those variables with P < 0.10 in the unadjusted analysis to adjust their effects for each other. The significant or suggestive results in the adjusted models are shown in italics

### Weighted pfPR and geometric mean parasite density by children’s age

**Fig. 4:**
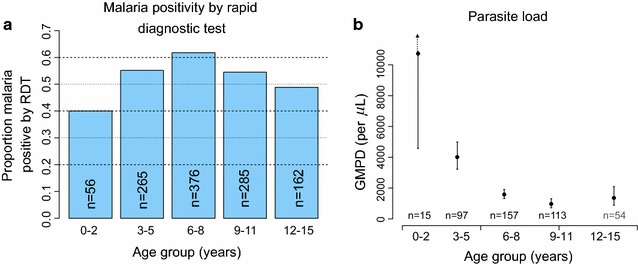
*Bar graphs* showing age-group patterns of weighted per cent *Plasmodium falciparum* parasite prevalence. Based on the RDT (**a**) and the GMPD/µL (**b**) among apparently healthy, microscopy-positive children enrolled between January 2011 and April 2015 from north-central and northwest Uganda. The unweighted number of participants in each age-group is shown. In **b**, the *solid circle* indicates the GMPD, the *lines* indicate the 95% CIs of the GMPD, except for the under 2 years old age group, where the upper boundary is beyond the *plotted points*. GMPD results were available on 436 of 441 microscopy positive subjects (see Table 2)

**Fig. 5:**
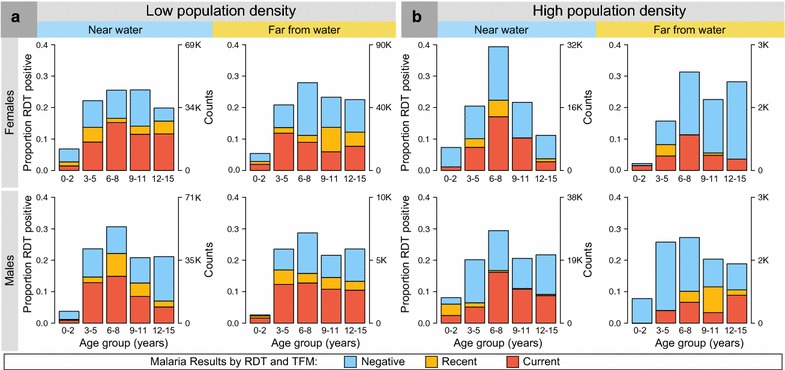
*Bar graphs* showing the weighted proportion of children (males and females) positive for *Plasmodium falciparum* parasite prevalence. Among apparently healthy children enrolled between January 2011 and April 2015 from north-central and northwest Uganda from low population density villages (**a**) and high population density villages (**b**). The *left y-axis* shows the proportion of children by pfPR results. The *right y-axis* shows the re-weighted population counts of the children represented by the sample in each stratum. The legend and colouring in *bars* shows pfPR categories based on both the RDT and TFM (see "Methods")

The weighted pfPR increased from 40.0% in children 1–2 years old to 61.8% in those 6–8 years old and decreased slightly to 48.9% in children 12–15 years old (Fig. 4a, b). Using logistic regression to model the weighted pfPR across five age categories that were previously defined in Emmanuel et al. (under 2, 3–5, 6–8, 9–11, 12+ years) [14], the age-specific ORs of pfPR were heterogeneous across these age categories. Compared to children under 2 years old, the ORs for pfPR peaked at 2.42 (95% CI 1.26–4.65) in children 6–8 years old and then decreased slightly to 1.43 (95% CI 0.62–3.31) in children 12–15 years old. There was a significant linear relationship between age and the weighted pfPR (*P*trend = 0.006) and a significant non-linear effect of age on pfPR (*P*trend = 0.004). These contrasting trends are consistent with the idea that the weighted pfPR increases with age as children become increasingly mobile with age and are more likely to be exposed to malaria infection. Conversely, increasing age is associated with increasing cumulative acquired malaria immunity and the chance that older children are more likely to successful resist new infection. Interestingly, these age trends were masked when pfPR across was modeled across three age categories (Table 3, *Pheterogeneity* = 0.185), highlighting the importance of using finer categories of age to capture rapid changes in the trends. Because BL epidemiology is characterized by male and rural predominance, the age-group specific patterns of pfPR were examined by sex and in high- and low-density villages, but no significant differences in age-specific patterns were noted by sex in the low-density (Fig. 5a) and high-density (Fig. 5b) villages.

Consistent with their asymptomatic status, GMPD among the 436 of 441 TFM-positive participants with quantitative data was comparatively low at 1805.5 parasites/µL (95% CI 1344.6–2424.3). However, GMPD was significantly and inversely correlated with age. GMPD was higher in children aged <5 versus ≥10 years old (5092.9 parasites/µL, 95% CI 2892.7–8966.8 versus 983.8 parasites/µL, 95% CI 472.7–2047.4; *P* = 0.001), consistent with the idea that immunity against malaria parasitaemia increases with age among highly exposed children.

### Association of pfPR with children’s characteristics

In unadjusted analyses, pfPR was positively associated with enrolment during the wet versus dry season (OR 2.35, 95% CI 1.26–4.40), and inversely associated with living in a sub-region where IRS was implemented versus living in a sub-region where IRS was not implemented (OR 0.26, 95% CI 0.14–0.46). Similar results were obtained when exposure to IRS was assessed using self-reported questionnaire information about whether a child lived or not in a house where IRS was applied in the past year (OR 0.28, 95% CI 0.15–0.53), confirming the validity of the ecological association between IRS and pfPR. The weighted pfPR was inversely associated with a child’s mother’s income ≥30,000 versus <30,000 USHS (OR 0.68, 95% CI 0.47–1.00) and positively associated with a child reporting outpatient treatment for malaria in the past 12 months versus no treatment (OR 1.73, 95% CI 1.1.0–2.72). However, sex, proximity of village to water, population density, region, and mother’s education level, sleeping under a mosquito bed net the previous night, or history of inpatient malaria treatment were unrelated to weighted pfPR (all *P* > 0.10). In multivariable analyses, including only those variables with *P* < 0.10 in the unadjusted analyses, the weighted pfPR remained significantly heterogeneous across the seven geographical sub-regions (*P* = 0.001). In addition, the weighted pfPR was inversely associated with a child’s mother income (OR 0.57, 95% CI 0.37–0.87) and with living in an IRS sub-region (OR 0.37, 95% CI 0.14–1.03), but positively associated with being enrolled during the wet season calendar months (OR 1.81, 95% CI 0.95–3.46). Similar patterns were observed when these analyses were repeated using pfPR defined based on only TFM or combined positivity TFM and RDT results.

## Discussion

The current study is the largest to present accurate granular baseline data about geographical and demographical patterns of asymptomatic malaria infection in healthy children living in a BL-endemic region. As expected, overall malaria prevalence in northern Uganda was high, consistent with the results from previous surveys from that region [20, 34, 35]. In agreement with the earlier studies, the weighted pfPR in the two neighboring regions studied was comparable. However, analysis of sub-regional patterns revealed 50% lower pfPR in sub-regions where IRS was employed compared to those where it was not applied, highlighting IRS as a major determinant of geographical patterns of malaria. However, substantial, significant geographical variation in pfPR was observed within IRS and non-IRS sub-regions, underscoring the contribution from other poorly understood geographical co-factors. This geographical variation, which had been masked by the overall regional-specific pfPR, included the entire spectrum of endemicity ranging from hypoendemic (1–10%), mesoendemic (pfPR 10–50%), hyper-endemic (pfPR 50–74%) to holoendemic (pfPR ≥75%) both within IRS and non-IRS sub-regions (Fig. 2c). The geographical variation in pfPR patterns observed here is likely correlated with the underlying variation in mosquito vector population relative to human hosts—the so called vector-to-host ratio [36], the prevailing vector species, e.g., *Anopheles gambiae* and *Anopheles funestus*, and their flying and feeding habits [37, 38]. Since recurrent immunological challenge from parasitaemia or malaria antigenaemia is the malaria exposure of interest in the study of BL, knowledge of these geographical patterns in pfPR can enable more precise evaluation of associations between malaria and BL through matching or adjustment on geography as a proxy for malaria pressure.

In addition to the environmental factors noted above, the geographical variation in the weighted pfPR might be due to genetic variation in prevalent genetic mutations like the sickle cell disease polymorphism that has been linked to malaria resistance [39]. Interestingly, the sub-region observed to have the highest pfPR in this study (sub-region 7; Fig. 1c) was also reported to have the highest prevalence of the sickle cell trait in northern Uganda (18–24%) [40]. This correlation is consistent with published literature suggesting positive selection of the sickle cell trait in areas of high malaria prevalence [41, 42]. This positive correlation could be due to a higher number of secondary malaria infections per infected person (also called the reproductive number of infection) among subjects with the sickle cell trait compared to those without the trait [43]. Specifically, because sickle cell trait carriers typically experience low GMPD infections [44] and low GMPD infections are more likely to persist and support higher rates of gametocyte production and lead to higher transmission rates in populations with a high frequency of the sickle cell trait [45]. Interestingly, Gouagna and colleagues [46] recently reported that parasites also may have adapted to be more efficiently transmitted to mosquitoes by persons with genetic variants in human haemoglobin gene as compared to non-carriers, thereby presenting evidence of parasite adaptation to host genetic mutations. This parasite adaptation to the human genetic mutations could potentially lead to stronger correlations between pfPR and the frequency of sickle cell trait in some areas.

Both pfPR and the GMPD were noted to vary with age, but in different ways. The weighted pfPR increased with age and peaked at ages 6–8 years, then declined slightly. Conversely, GMPD peaked quickly in children aged under 2, 4–6 years before peak pfPR age, then declined rapidly. These contrasting pfPR/GMPD patterns suggest that children are exposed to malaria infection at all ages and infection is usually successfully established until they reach 6–8 years, when the risk of new infection decreases. The GMPD patterns, which decrease rapidly with age, suggest that high parasite counts are experienced mostly at the young ages when children still lack immunity, but infections at older ages occur in the context of immunity to malaria, which suppresses parasitaemia [47]. Given the interest in malaria infection as a risk factor for BL, the observation that GMPD peaks about 4–8 years earlier than BL [14] argues against high GMPD being the insult that triggers the onset of such a rapidly growing tumor [16]. Rather, the occurrence of BL in older children, who are more likely to have lower parasitaemia (Fig. 4b), suggests that recurrent low-count afebrile parasitaemia, probably due to novel allelic variants that cause break-through infections in children with established immunity [48, 49], may be the underlying malaria antigenic insult that triggers the onset of this rapidly growing tumor [50]. If so, examination of immune responses to malaria or allelic variants in patients with BL and well-matched controls could reveal both evidence of strong exposure to malaria, strong protective immune response against malaria, and infection with multi-clonal parasites in children with BL.

The observation of an inverse correlation between pfPR and the use of IRS agrees with results from national malaria indicator surveys conducted in Uganda [21]. Similar to those surveys, the current study also found that mosquito bed nets, which are widely recommended and distributed [51], appear to have had a negligible impact on geographical or individual pfPR patterns in this region. This lack of impact of mosquito bed nets on pfPR in this study, which agrees with reports by others in the region [52], may, in part, be due to misclassification of responses about bed net use or to their inconsistent and/or improper use. However, mosquito bed nets only suppress mosquito populations around bed areas, which may not lead to community-wide suppression of the vector-host ratio like IRS. More worrying, the lack of impact could be an indicator of emerging pyrethroid resistance in this region as has been reported in some studies [53]. If so, the lack of impact of mosquito bed nets on geographical patterns of pfPR should be evaluated more carefully to understand its basis and to exclude more serious reasons such as emerging pyrethroid resistance in regional mosquito populations.

The strengths of the current study include selecting a regionally representative, population-based sample of children to generate accurate regional and sub-regional statistics of pfPR in a BL-endemic area. The use of RDT, which is more sensitive than TFM and provides information about population prevalence of malaria infection over a three- to four-week period [33] is a strength. The finding of similar patterns of pfPR when using results defined by TFM or both TFM and RDT suggests that the patterns reported here are likely valid. Only 1.1% of participants had false negative RDT results, which is similar to prior reports [54]. This percentage is negligible for descriptive studies of geographical and demographical patterns, but the molecular reasons for false negative results, which may be due to deletions of *pfhrp2* or *pfhrp3* genes that code for the RDT antigens [54], need careful evaluation to preserve the value of these tests for clinical and epidemiological studies. The limitations of this study include relying on recall for information about history of outpatient or inpatient malaria treatment, which is subject to recall bias. Review of health records at health centers was considered to strengthen the results, but the quality of records at health centres in rural Uganda was found to be unreliable. Another limitation is the classification of wet and dry season based on average rain patterns during the study period, hence there might be some misclassification, which would attenuate differences.

## Conclusions

The study confirmed high but geographically and demographically heterogeneous malaria prevalence in BL-age children residing in northern Uganda. The finding that the peak age for pfPR and BL are comparable, while GMPD peaks 4–8 years earlier, raises the hypothesis that recurrent asymptomatic malaria exposure in children with established malarial immunity may be the underlying malaria antigenic insult associated with the onset of BL. Understanding the geographical patterns of malaria in this BL-endemic area will pave the way for a more granular examination of the malaria/BL associations in follow-up studies using molecular and immunological methods to measure the malaria response in patients with and without BL.

## Additional file

**Additional file 1.**
 Appendices I and II.

## Abbreviations

pfPR: *Plasmodium falciparum* prevalence
*pfhrp2*: *Plasmodium falciparum* histidine-rich protein 2
*pfhrp3*: *Plasmodium falciparum* histidine-rich protein 3
GMPD: geometric mean parasite density
OR: odds ratio
CI: confidence interval
AEIR: annual entomological inoculation rate
BL: Burkitt lymhoma
IRS: indoor residual spraying
EA: enumeration area
UBOS: Uganda Bureau of Statistics
TFM: thick film microscopy
WBC: while blood cell
RDT: rapid diagnostic test
pLDH: pan lactate dehydrogenase
USHS: Ugandan shillings
US$: United States dollar
