# Supplementary material for: Age and geographic patterns of Plasmodium falciparum malaria infection in a representative sample of children living in Burkitt lymphoma-endemic areas of northern Uganda
Source: Malar J. 2017 Mar 20;16:124. doi: 10.1186/s12936-017-1778-z (PMC5360076; doi:10.1186/s12936-017-1778-z)
Supplement: Supplementary file 1 — Additional file 1. [file 12936_2017_1778_MOESM1_ESM.pdf]

# Appendices

**Age and Geographic Patterns of *Plasmodium Falciparum* Malaria Infection in A Representative Sample of Children Living in Burkitt lymphoma-endemic Areas of Northern Uganda, Maziarz *et al*.**

## Appendix 1. Calculation of sampling weights

***Primary sampling weights***. In the first stage of sampling, 100 Enumeration Areas (EAs), which constituted the primary sampling units (PSUs), were selected from the following four strata: near water (wet) and low population density (rural) (WR), far from ambient water (dry) with low population density (DR), near ambient water and high population density (urban) (WU), far from ambient water with high population density (DU). Of the 100 EAs, 12 were selected in the pilot phase of the study, followed by sampling of additional 88 EAs for the main study. Specifically, we sampled (4, 4, 2, 2) pilot EAs from (WR, DR, WU, DU) respectively, and (30, 30, 14, 14) main EAs from (WR, DR, WU, DU), respectively. Thus, the primary sampling weights (fraction on the left) for the PPC EAs were

$$\frac{\text{\# of EAs in strata}}{\text{\# of EAs selected in pilot phase}} \; \frac{\text{\# selected in pilot phase}}{\text{\# selected total in pilot and main phases}}$$

The weights were (605/4*4/34, 115/4*4/34, 227/2*2/16, 35/2*2/16) for (WR, DR, WU, DU) respectively.

For the MPC EAs, the primary sampling weights were:

$$\frac{\text{\# of EAs in strata}}{\text{\# of EAs selected in main phase}} \; \frac{\text{\# selected in main phase}}{\text{\# selected total in pilot and main phases}}$$

The weights were (601/30*30/34, 111/30*30/34, 225/14*14/16, 33/14*14/16) for (WR, DR, WU, DU) respectively. Thus, after combining the 12 pilot and 88 main EAs the adjusted primary weights represent the total number of EAs in a given strata.

***Secondary sampling weights.*** One village was sampled per EA. Thus, the secondary sampling weight is the number of eligible villages in a given EA.

***Tertiary sampling weights.*** At this level children were sampled from a list of all eligible in a given village, conditioning on age group at time of census (0-<3, 3-<6, 6-<9, 9-<12, 12+ years of age) and sex. The number of eligible children in a given age group was adjusted for infant and child mortality as estimated by the UBOS in the 2006 survey [1]. According to UBOS, infant mortality (mortality in the first year of life) was 76 out of 1000 live births, and child mortality (mortality between age one and five) was 67 per 1000 live births. The lowest two age groups for sampling weights were 0-<3 and 3-<6, thus the mortality rates were approximated for those age groups. The mortality rate for children aged 2-<6 was assumed to be constant. Then, the mortality rate for children in the 0-<3 age group was approximated as the infant mortality rate plus the average annual child mortality rate over two years: (76 + 67/4*2)/1000 = 0.1095. The mortality rate for children in the 3-<6 age group was approximated by the average annual child mortality rate over a three-year period: (67/4*3)/1000 = 0.05025. The number of eligible children in the sampling weight calculation was multiplied by (1000-76 - 67/4*2)/1000 = 0.8905 for the 0-<3 age group and by (1000-67/4*3)/1000 =0.94975 for the 3-<6 age group. Thus, the tertiary sampling weights were equal to the number of eligible children in a given sex and age group (adjusted for mortality in the lowest two age groups) divided by the number selected in that sex and age group in a given village. For the subjects who were originally sampled in the pilot villages (n=113) tertiary sampling weights were additionally multiplied by the number of eligible pilot controls divided by the number of selected pilot subjects in a given sex and age group.

***Final sampling weights**:* The final sampling weight for each child was the product of their primary, secondary and tertiary weights. The coefficient of variation of the final weights was 1.27. A histogram of the weights is shown in Supplemental Figure 1.

**Supplemental figure 1: Distribution of sampling weights**

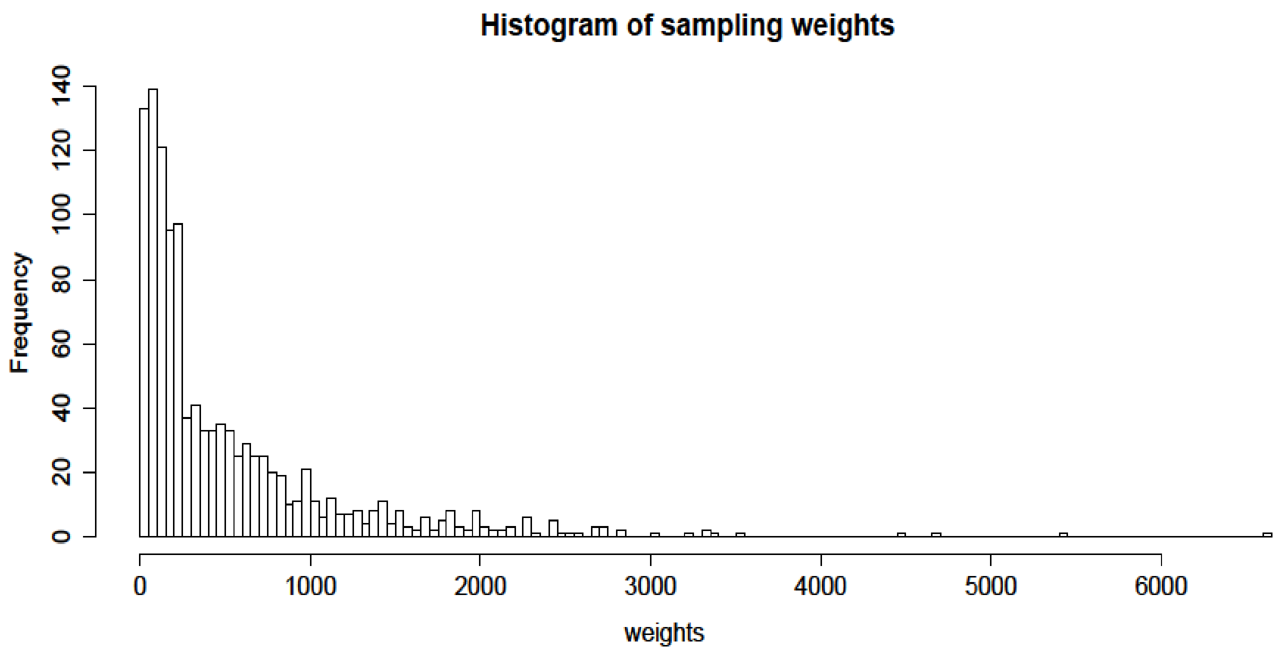

# Appendix 2. Computational details and R code used for the analysis

### Sampling design setup

The multistage sampling design and the computation of the sampling weights (denoted by the variable "weights1234" below)are described in detail in Figure 1 in the main paper and Appendix 1. In brief, the sampling design of our study is defined by strata (4 strata, referred to as "sampling.strata" from here on), enumeration areas (EAs), which are the primary sampling units (PSUs), and village. In the computations, we accommodate clustering at the village level, noting that there is a one-to-one correspondence between PSU and village.

The 'survey' package (version 3.31) in R [2, 3] was used for all the weighted analyses. In this package the design is incorporated by

```
d.svy <- svydesign(ids = ~village,
            weights = ~weights1234,
                              strata = ~sampling.strata,
                              data = d)
```

The svydesign object d.svy is passed to all the functions that follow. The presentation below describes the R functions used for each Table.

1. **Weighted contingency tables: totals and percentages (Table 1)**

```
svytotal(~interaction(malaria_RDT, malaria_microscopy),
       design=d.svy)
```

The percentages are the counts in each cell divided by their sum.

2. **Weighted proportions and odds ratios (ORs) and 95% confidence intervals (Cis) (Table 3, Supplementary Table 1)**

To obtain weighted proportions we used the function:

```
svyby(~outcome by =~covariate, FUN = svymean, design=d.svy)
```

This function was called for each covariate in the rows of the table separately. Outcome for Table 2 is “malaria positivity by RDT”, for Supplementary Table 2 it is “microscopy” and “joint RDT and microscopy results”.

```
svyglm.object <- svyglm(outcome ~ covariate,
                        family = quasibinomial(),
                        design=d.svy)
```

was used to compute ORs and the 95% CIs for each covariate in the table separately.

```
svyglm.object <- svyglm(outcome ~ covariate1 + covariate2 + …,
                        family = quasibinomial(),
                        design=d.svy)
```

where "covariate1 + covariate2 + …" lists all covariates in the model.

P-values for the association of a covariate with the outcome for unadjusted and adjusted logistic models were calculated using svyglm object from the unadjusted or adjusted models using:

```
regTermTest(svyglm.object,
      test.terms = ~ covariate,
      method = 'Wald')
```

3. **Weighted proportions stratified by sampling strata (Table 4, Supp. Tables 3-5)**

```
svyglm.object <- svyby(~ outcome,
            by = covariate + sampling.strata,
            FUN = svymean,
                        design=d.svy)
```

This function was called for each covariate separately.

4. **P-values for heterogeneity within sampling strata (Table 4, Supplementary Table 4)**

```
svyglm.object <- svyglm(
   outcome ~ covariate + strata.water + covariate:strata.water,
   subset = one of the levels of population density strata,
   family = quasibinomial(),
   design=d.svy)

regTermTest(svyglm.object,
            test.terms = ~ covariate:strata.water,
            method = 'Wald')
```

where strata.water is a strata variable based on distance of the village from water.

5. **P-values for the overall test of heterogeneity (Table 4, Supplementary Table 4)**

```
svyglm.object <- svyglm(
  outcome~covariate+sampling.strata+covariate:sampling.strata,
  family = quasibinomial(), design=d.svy)

regTermTest(svyglm.object,
        test.terms = ~covariate:sampling.strata,
            method = 'Wald')
```

6. **ORs (95% CIs) and p-values for a model assessing association of RDT with age (Supplementary Table 2)**

To assess age effect in more detail we fit a model with a linear and quadratic term to age, coded using the midpoints of 0-2, 3-5, 6-8, 9-11, 12-15 year intervals using:

***Model with a linear and quadratic effect of age on RDT positivity:***

```
svyglm.object.m2 <- svyglm(
      outcome ~ age.group.midpoint + I(age.group.midpoint^2),
      family = quasibinomial(),
      design=d.svy)
```

with age.group.midpoint coded as a numeric variable of midpoints of age groups listed in the table.

The overall p-value for the age effect in model with a linear and quadratic effect of age was computed from :

```
regTermTest(svyglm.object.m2,
        test.terms = ~ age.group.midpoint+I(age.group.midpoint^2),
    method = 'Wald')
```

## References


1. 2002 Uganda Population and Housing Census. Kampala: Government of Uganda (Uganda Bureau of Statistics). 2007.

2. Lumley, T. (2010). Complex surveys: A guide to analysis using R (Wiley series in survey methodology). Hoboken, N.J.: John Wiley.

3. Lumley, T. (2016). Reference manual for the 'survey' package in R. Retrieved from: https://cran.r-project.org/web/packages/survey/survey.pdf
